# Supporting adolescent emotional health in schools: a mixed methods study of student and staff views in England

**DOI:** 10.1186/1471-2458-9-403

**Published:** 2009-10-31

**Authors:** Judi Kidger, Jenny L Donovan, Lucy Biddle, Rona Campbell, David Gunnell

**Affiliations:** 1 Department of Social Medicine, University of Bristol, Canynge Hall, 39, Whatley Road, Bristol BS8 2PS, UK

## Abstract

**Background:**

Schools have been identified as an important place in which to support adolescent emotional health, although evidence as to which interventions are effective remains limited. Relatively little is known about student and staff views regarding current school-based emotional health provision and what they would like to see in the future, and this is what this study explored.

**Methods:**

A random sample of 296 English secondary schools were surveyed to quantify current level of emotional health provision. Qualitative student focus groups (27 groups, 154 students aged 12-14) and staff interviews (12 interviews, 15 individuals) were conducted in eight schools, purposively sampled from the survey respondents to ensure a range of emotional health activity, free school meal eligibility and location. Data were analysed thematically, following a constant comparison approach.

**Results:**

Emergent themes were grouped into three areas in which participants felt schools did or could intervene: emotional health in the curriculum, support for those in distress, and the physical and psychosocial environment. Little time was spent teaching about emotional health in the curriculum, and most staff and students wanted more. Opportunities to explore emotions in other curriculum subjects were valued. All schools provided some support for students experiencing emotional distress, but the type and quality varied a great deal. Students wanted an increase in school-based help sources that were confidential, available to all and sympathetic, and were concerned that accessing support should not lead to stigma. Finally, staff and students emphasised the need to consider the whole school environment in order to address sources of distress such as bullying and teacher-student relationships, but also to increase activities that enhanced emotional health.

**Conclusion:**

Staff and students identified several ways in which schools can improve their support of adolescent emotional health, both within and outside the curriculum. However, such changes should be introduced as part of a wider consideration of how the whole school environment can be more supportive of students' emotional health. Clearer guidance at policy level, more rigorous evaluation of current interventions, and greater dissemination of good practice is necessary to ensure adolescents' emotional health needs are addressed effectively within schools.

## Background

Rates of clinical and subclinical emotional health problems during adolescence [1-3], and the multiple detrimental health and social outcomes with which they are associated, such as suicide attempts, substance misuse, educational underachievement, unemployment and long-term psychiatric disorders [4-6], have been a cause of concern for some time. Many emotional disorders have their onset in adolescence [6], and a marked increase in prevalence occurs from the middle to late teenage years [7,8]. Further, many teenagers who experience emotional health problems fail to receive help from appropriate services [9,10]. Such evidence provides strong arguments for community based interventions to support emotional health in early adolescence, and prevent or reduce the onset of disorders.

Several examples exist of classroom-based programmes in schools that focus on emotional health, and these fall roughly into three groups. The first group aims to prevent or reduce emotional disorders such as depression and anxiety, by developing coping skills commonly used within psychiatry [11-13]. A number of reviews, while acknowledging the potential value of such interventions, conclude that evidence for their long-term effectiveness remains limited [14-17]. The second group comprises interventions that focus more on the improvement of knowledge and understanding regarding emotional health related issues, with a view to addressing concerns such as self-harm and suicide, negative attitudes towards emotional disorders and adolescents' unwillingness to seek help [9,18,19]. A small number of such interventions have been evaluated and found to be effective [e.g. [9,18,20]], but again evidence from suitably high quality evaluations is lacking, and there is a need to establish both long-term effectiveness, and whether changes in knowledge and understanding lead to the desired behavioural change. The third group of programmes focuses more on promoting positive emotional health, sometimes called 'emotional intelligence' or 'emotional literacy' [21]. For example the government-led initiative SEAL (Social and Emotional Aspects of Learning) in England [22], and SEL (Social and Emotional Learning) programmes developed in the USA [23], both aim to develop emotional and social skills in order to promote emotional health, improve behaviour, and support academic learning. While the evaluation of SEAL is still ongoing, SEL has been found to be effective in social and emotional skills development, increasing positive social behaviour, increasing academic attainment, and reducing emotional distress and conduct problems [23].

A concurrent development to classroom-based work has been an interest in 'whole-school' approaches, which is in keeping with a wider move towards a focus on the whole school when implementing health interventions [24]. Such programmes often aim at promoting emotional health rather than preventing emotional disorder [25], and take a holistic approach to this, considering all aspects of school life including policies and procedures, ethos, and partnerships with external agencies and parents to better support students' emotional health [25,26]. This model is not necessarily in opposition to classroom-based programmes, but is seen as providing an important backdrop against which activities such as emotional health lessons and support services for those who need them will be more effective [27]. Justification for such an approach can be found in the reported association between emotional health and factors related to school life such as academic success, peer relationships and school connectedness [28-31]. Further, there is growing evidence that whole-school approaches focusing on promoting good emotional health are more effective than stand alone classroom-based interventions that aim to prevent emotional disorder [27,32], although more evaluations, particularly beyond the elementary school years, are still needed in this area.

Within England, the significance of school settings for addressing the emotional health of all students is emphasised in key policy documents and initiatives [22,33,34]. Current thinking appears to support an emphasis on improving emotional health rather than preventing disorder, and on addressing whole-school issues such as school climate, provision of support services and the professional development of teachers, alongside curriculum based work [22,35]. A report by England's educational inspectorate Ofsted in 2005 identified several elements that characterised schools that were successfully promoting emotional health, including a caring ethos, teacher training in how to promote emotional health, and good communication with external agencies and parents [36]. However, the report concluded that very few secondary schools (age range 11-16) contained these elements or were adequately supporting emotional health, despite the national policy drive in this area [36].

Alongside the need for stronger evidence regarding what works in school-based emotional health initiatives, there is also a need to establish how far the range of potential interventions match what young people themselves say they want or need. Involving the perspectives of teenagers in the design of interventions is an important way of giving them a voice in issues that affect them, as well as helping to ensure that provision is as appropriate as possible [37]. However, interventions to date have often not taken their views into account sufficiently, resulting in a mismatch between what programmes have targeted and what young people say about their own lives [38]. Key findings from previous research on adolescents' views are that they struggle to define terms such as mental health and depression, that talking to someone is seen as an important source of support although many individuals have anxieties about seeking help from adults, and that aspects of school life such as workload and being bullied can be a significant cause of distress, but that schools are also viewed as potential sources of information and support [37,39,40]. An even smaller number of studies have explored teachers' views regarding the needs of students in relation to emotional health, and what schools could or should be doing to support those needs. Previous findings indicate that, although teachers may accept emotional health support as an integral part of their role as educators of young people, they often feel burdened by the emotional health needs of their students, and feel they lack knowledge in how to provide support, or discuss emotional health issues with them [41-43].

This paper reports findings from a study that sought to increase our understanding of both staff and students' perceptions of school-based emotional health. Specifically, the study aimed to examine the views of staff involved in emotional health work and students regarding current school-based emotional health provision, how far this meets the needs they identify and what they would like to see schools do in the future. The long term aim of this work is to develop a school-based intervention to improve the emotional health and well-being of secondary school children.

As emotional health can be addressed in a variety of ways in school, the notion of emotional health work was kept deliberately broad to ensure that all aspects that participants viewed as important were captured. It therefore included anything that had the potential to improve emotional health or reduce emotional difficulty, such as extra-curricular activities, support from counsellors or other school-based staff, and the delivery of lesson content that explicitly related to emotional health. The term 'emotional health and wellbeing' (EHWB) was used throughout the data collection as, since its introduction as a concept through the National Healthy Schools Programme [34], it has become one of the terms most commonly used within English educational settings to refer to all aspects of emotional health. The British Educational Research Association's Revised Ethical Guidelines for Educational Research 2004 were followed in the design and conduct of the study, and the study was approved by the University of Bristol's Social Sciences and Law Faculty Ethics Committee.

## Methods

A mixed methods approach was used to enable us both to quantify the level of emotional health provision in English secondary schools and to use survey responses to inform the sampling of schools for in depth qualitative investigation of student and teacher views about current provision and areas for development. At the beginning of the study, a postal questionnaire enquiring about EHWB activities was sent to a random sample of 296 secondary schools in England. The questionnaire was sent to the headteacher, with the request that they pass it on to the member of staff best placed to give an overview of EHWB provision within and outside the curriculum. Questions asked how high a priority EHWB was in the school (choice of five responses from very low to very high), whether a list of EHWB topics were taught in lessons, and if a range of other EHWB related activities or support were provided such as on-site counselling, links with external agencies, relevant policies and teacher training (respondents were asked to tick yes/no in each case).

Qualitative methods were used to address the main study question, that is staff and students' views regarding current and potential emotional health provision in schools. This approach was taken due to its emphasis on participants' perspectives and interpretation of the world [44], and its ability to allow in-depth and unanticipated findings to emerge, which was deemed advantageous due to the under-explored nature of this area.

### Sample selection

The questionnaire sample was obtained from EduBase - a register of all educational establishments in England and Wales  - who provided a random sample of schools from a sampling frame of all non-fee paying secondary schools in England. The sample was made up of two schools from every Local Education Authority area (n = 296), and stratified for deprivation of catchment area (using percentage of students eligible for free school meals as a proxy measure). This stage of the process required a random sample, as the aim was to gain a representative picture of what was happening in schools, and to create a representative sampling frame for the qualitative study.

Schools were selected for the qualitative study purposively, to ensure those that were included covered the range of amount and types of emotional health support seen in the national picture. Three factors were taken into account in sampling schools: a) extensiveness of EHWB curriculum coverage and support (as reported in the questionnaire) b) geographical position of the school c) percentage of pupils eligible for free school meals (an indicator of parental income) relative to the national average. The aim was for the final sample to represent different combinations of these three factors, as it was hypothesised that they may have a bearing on student and staff opinions about EHWB need and provision [45].

When approached, three of the schools declined to take part due to lack of time or in one case, impending closure, and they were replaced by schools that matched them as far as possible on level of activity, free school meal eligibility and location. A summary of the final qualitative sample is given in table 1.

**Table 1:** Summary of schools that participated in the study

**School**	**Region**	**Free School Meal Eligibility ** **(%)**	**EHWB Activity**	**Focus Groups Undertaken**		**Staff Interviewed**
				**Year 8** **12/13 yrs**	**Year 9** **13/14 yrs**	

1	SW	22.9	Low	1 male 1 female		PSHE coordinator
						
2	SW	6.0	High	1 male 1 female		Head of year
						
3	SW	27.6	High	1 male 1 female	1 male 1 female	Assistant Principal Learning Support Manager
						
4	SW	4.2	High	1 male 1 female	1 male 1 female	Head Key Stage 3
						
5	NW	3.5	Low	1 male 1 female	1 male 1 female	Head Key Stage 3
						
6	London	17.5	Low	1 male 1 female	1 male 1 female	Learning Support Manager Psychologist 3 Teaching assistants
						
7	Midlands	21.3	Low	1 male	1 male 1 female	2 Learning Mentors
						
8	NE	26.4	High	1 male 1 female	1 male 1 female	SEN Coordinator Head of Year

Notes

1. Percentage of pupils eligible for free school meals was taken as a proxy measure of deprivation of school catchment area. National average for England is 14%.

2. See table 2 for fuller description of staff roles.

### Qualitative Data Collection

#### Staff interviews

Initial contact with each of the eight schools was made through a deputy or assistant head whose job included responsibility for pastoral care, and this was the individual who agreed to the school taking part. They then identified another member of staff who was responsible for some aspect of EHWB work in the school to liaise with the study team. The nature of this contact's role varied, but included teachers responsible for coordinating the personal, social and health education (PSHE) programme within the curriculum, staff heading up a support unit for students at risk of exclusion, and staff who managed the school's special educational needs provision. Each contact was invited to be interviewed, and to suggest one or two colleagues also involved in delivering work that supported EHWB, who might be willing to be interviewed. As there is a great deal of variety from school to school regarding which staff are involved with EHWB support, this method of identifying two or three key staff members to approach was deemed most appropriate, and generated a wide range of interviewees (see table 2), which helped build up a picture of the different ways in which schools support emotional health. The interviews were held on school grounds during the school day, either in private offices or empty classrooms. Written information was given to each potential interviewee about the aims of the project, and the procedures to ensure confidentiality and anonymity that would be adhered to, and written consent obtained from those who agreed to take part.

**Table 2:** Description of Staff Interviewees

**Job Title**	**Number of Participants**	**Job Description**
*Teaching Staff*		
Head of Key Stage 3 (11-14 year olds)	2	Senior teacher. Role involves teaching but also managing other teachers and coordinating teaching and support for first three years (grades) of secondary school.
		
Head of Year	2	Senior teacher. Role involves teaching but also managing other teachers and coordinating teaching and support for particular year (grade).
		
PSHE Coordinator	1	Role involves teaching, but also coordinating the Personal Social and Health Education curriculum, which is where most teaching about health takes place.
		
Teaching Assistant	3	Role involves supporting teachers within the classroom, often by offering one to one support or support to small groups of students with particular needs.
		
*Other Positions*		
Assistant Principal	1	Member of senior management team. No longer involved in teaching, but manages particular aspects of the school. This interviewee was responsible for the pastoral care in the school among other things.
		
Learning Mentor	2	Employed to provide one to one support to students to improve attainment and reduce exclusions. One of these interviewees had previously been a teacher.
		
Learning Support Manager	2	Responsible for the Learning Support Unit, which provides short term teaching and support to vulnerable students and those at risk of exclusion. One of these interviewees had previously been a teacher.
		
Special Educational Needs Coordinator	1	Responsible for the support offered to students with special educational needs, for example those that arise in relation to learning difficulties or mental health problems.

#### Student Focus Groups

In each of the eight schools, focus groups were conducted with students in years 8 (12-13 year olds) and 9 (13-14 year olds). There were two main reasons for using this method. Kitzinger writes that "group processes can help people to explore and clarify their views in ways that would be less easily accessible in a one to one interview" [[46] p300], therefore it was hoped that a group discussion might enable participants to develop ideas and co-construct knowledge around issues that they may not spend a lot of time reflecting on in their day to day lives. Secondly, the stigma that can surround emotional distress and disclosure of emotions makes discussion of emotional health a potentially sensitive topic [47]. Although discussing sensitive issues in groups can create difficulties in terms of certain views being stigmatised or remaining unexpressed, it was felt that the power differential in a one to one interview between an adult researcher and a teenager might make such discussions even less comfortable for the participants. Therefore the focus group was chosen in the hope that it would help break the ice for the more inhibited participants, and provide a supportive setting in which they could express their views [46]. Because of this concern about the potentially sensitive nature of the subject matter, the topic guides only asked about the needs of teenagers in general, they did not ask about personal experiences, which are likely to be more appropriately explored within an interview setting. In addition, the groups were made up of friendship pairs, that is half of the focus group participants were selected by form teachers or heads of year, and then each chosen participant was invited to bring a friend with them, in an attempt to ensure a supportive atmosphere, a technique which has been used elsewhere [48].

Most writers suggest that homogeneity in focus groups can be an important way to reduce inhibitions and ensure participants feel comfortable enough to share their views and feelings [49,50]. Evidence that boys and girls may have differences in emotional health needs and favoured coping strategies [2,51], and that both boys and girls can find it difficult to speak openly in front of the other gender [52] led to a decision to make the focus groups single sex. Single-sex focus groups to explore health related topics where gender differences exist has been used elsewhere [48].

The staff selecting the participants were asked to provide a range of students in terms of confidence and academic ability. Information leaflets were provided to each student and information letters were sent to their parents or guardians, both of which outlined the purpose of the study, what would be done with the data, and how confidentiality and anonymity would be assured. Written consent to participate was gained from both student and parent or guardian. The focus groups were held on school grounds during lesson time, in empty classrooms or offices.

In both the focus groups and the interviews, a list of topics was followed, but with sufficient flexibility to allow participants to raise and focus on the issues that they identified as important. The staff interview topic guide is shown in table 3, and the focus group topic guide is shown in table 4.

**Table 3:** 

**Staff Interview Topic Guide**
A. GENERAL Their role in relation to EHWB provision What they understand by the term EHWB Students' main needs in this area Teachers' main needs in this area Is it important for schools to deliver EHWB? B. WITHIN THE CURRICULUM Anything that is covered now, how and by whom What other things they would like to see covered, how and by whom How they feel about teaching EHWB and related topics Barriers and facilitators to the school covering EHWB in the curriculum C. OUTSIDE THE CURRICULUM Anything the school provides to enhance the emotional health and wellbeing of pupils What they would like to see that is not currently provided Anything this school provides to enhance the emotional health and wellbeing of teachers What they would like to see for teachers that is not currently provided What services are provided for students experiencing emotional difficulty? Are there things that could be provided to help students deal with emotional difficulties that are not currently? Barriers and facilitators to the school supporting EHWB outside the curriculum Links with external agencies D. FINAL COMMENTS What is their understanding of a whole-school approach to addressing EHWB? Do they think this school takes a whole-school approach? What would help this school address EHWB more effectively? Anything else they want to say about EHWB in this school or in schools generally

**Table 4:** Student Focus Group Topic Guide

A. OUTSIDE THE CURRICULUM
What makes young people feel good or happy?
Are there things in school that help students feel good or happy?
What else could be done or changed in school to make students feel good or happy?
What sort of problems do people your age have?
What difficult feelings do people your age have?
Do you think teachers are aware of the sorts of problems that young people face?
Are there things this school does or provides that help students who are having problems or difficult feelings?
What do young people do if they have a problem/difficult feelings?
Are there people in school you can go to if you have those problems/feelings?
Are there things that could be changed/set up in schools that would help people who were having problems/difficult feelings?

B. IN THE CURRICULUM
Do you have lessons on emotional health and wellbeing?
IF YES: how many, topics, who teaches, what they do, what they like/dislike about the lessons, whether useful or not
IF NO: would you like lessons on this?
Does the subject emotional health and wellbeing come up in other lessons?
Is there anything else you want to say about what this school could do, to make it a better place for students to be?

### Data Analysis

All interviews and focus groups were audio-recorded and transcribed. The focus groups and interviews were analysed separately, using constant comparison techniques common to qualitative research [53]. Transcripts were scrutinised for emergent themes, and relevant sections of text were coded and grouped together according to those themes. Where the original theme identified was quite broad, it was broken down into sub themes, as further relevant sections of text were identified. After the first transcript had undergone analysis in this way, the identified themes were compiled into a coding frame. Each new transcript was then analysed for themes, which were compared against this initial coding frame. Those themes that did not fit the existing frame were either added as new themes, or were used to expand and modify existing themes, until all data had been accounted for. As this process continued, conceptual themes were developed from the initial descriptive themes, and previously analysed transcripts were then re-examined to identify any other sections of text relevant to these new themes. To explore similarities and differences across groups on key themes, matrix displays were used [54], in which the relevant sections of text from each focus group were drawn together in a table format for easy comparison. Descriptive accounts of each focus group were constructed, to ensure that any within group differences regarding the themes identified were acknowledged and included in the analysis.

Initial focus group and interview transcripts were analysed independently by two members of the research team, to check the reliability of the coding frame [55]. Inter-rater reliability was good, with no disagreements arising over the definition of themes that emerged from the text, or regarding the themes that each section of text related to. Some differences between raters arose as to whether the themes they identified were new or were subthemes of existing themes. For example one member of the team included depression as a subtheme of negative feelings experienced by teenagers, whereas another identified depression as a new main theme. Where such differences arose, the theme in question was included as a main theme, but was cross referenced with the existing relevant theme.

## Results

### Questionnaire survey

Questionnaires were returned from 75 schools (25.3%). This low response rate may have been due the questionnaire not reaching the right member of staff - all questionnaires were sent to the headteacher with the request to pass it to the most appropriate person. A further explanation may have been the feeling among schools that they receive too many surveys, which has led to a decline in responses to research surveys and government audits in recent years, with the majority conducted since 2000 having a response rate of less than 50% [56]. Responders and non-responders were compared using chi-square tests on dimensions that were hypothesised to affect emotional health provision in schools: size, free school meal eligibility, SATS results (national examinations taken at age 14) and religious affiliation. Differences found on these dimensions were slight (table 5). A phone survey of a subset of the questions was conducted with 17 non-responding schools. It revealed that they were less likely to view EHWB as of high importance (47.1%) compared to those who had responded (68.6%), and were less likely to provide an onsite counsellor (47.1% compared to 80%) and a drop-in health service (68.8% compared to 84.5%). Therefore it may be that the schools that did respond were those who were most interested in EHWB provision.

**Table 5:** Comparison of responders and non-responders on key variables

**Key Variables**	**Responders** **N (%)**	**Non-responders** **N (%)**
Below average size	30 (42.25)	118 (52.7)
Below average free school meal eligibility	38 (53.5)	114 (50.9)
Above average SATS results	37 (52.1)	105 (46.9)
Religious affiliation	26 (36.6)	71 (31.7)

Notes

1. Although all state schools receive local authority funding, follow the national curriculum, and are inspected by the national childcare inspectorate Ofsted, two types (voluntary-controlled and voluntary-aided) work in partnership with at least one charitable foundation, which is often a religious organisation. This organisation typically owns the school buildings, and may appoint some members of the governing body, as well as having some influence over the ethos and religious teaching in the school.

Respondents to the survey were generally deputy headteachers responsible for pastoral care, Personal, Social and Health Education (PSHE) coordinators, staff in charge of a whole year (grade) or staff responsible for student support. The proportions of responding schools that provided key emotional health and wellbeing activities are shown in table 6. While most respondents covered bullying and improving emotional health in lessons, only around one third covered self-harm or depression. At least 80% provided an on-site counsellor, a drop-in health service, a peer support service and support groups for vulnerable students. Less than half provided any training for staff members involved in delivering EHWB work, and only 26.5% provided emotional health training for teachers in general.

**Table 6:** Proportion of schools providing key EHWB activities

**Provision/coverage**	**N (%)**
*EHWB topics covered in the curriculum*	
Improving emotional health	51 (78.5)
Bullying	65 (97.0)
Strategies for dealing with emotional distress	44 (66.7)
Reducing the stigma of emotional distress	33 (44)
Causes, symptoms and treatment of depression	22 (35.5)
Self-harm	21 (32.8)
*Extra-curricula EHWB activities*	
On-site counsellor	56 (80.0)
Drop-in health service	60 (84.5)
Peer support service	58 (82.9)
Support groups for vulnerable students	64 (90.1)
Training for teachers delivering EHWB lessons and activities	34 (47.8)
Training for teachers in general about emotional health	18 (26.5)
School policy that focuses on emotional health	33 (47.8)
Regular contact with relevant health services e.g. CAMHS	56 (82.4)

### Focus groups and interviews

Twenty seven focus groups (154 students) ranging from four to eight students in size, and 12 individual or paired staff interviews (15 individuals) were conducted in total (table 1). The same number of focus groups and staff interviews were not completed in all eight schools, due to the amount of time each school was able to give to the study. Participants' perceptions of the emotional health provision within their schools led to identification of three overarching categories: (i) EHWB in lessons (ii) support for students experiencing emotional difficulty, and (iii) wider aspects of the school environment. The key themes that emerged with regard to each of these categories are presented, with illustrative quotes.

#### EHWB in lessons

The main themes within this category were a lack of EHWB material in lessons, the inadequacy of the EHWB teaching that did exist, a desire for external experts to teach EHWB topics, concerns regarding the stigma of discussing emotions or of experiencing emotional difficulty and the potential for learning about EHWB through other, non-health lessons.

The general feeling among students was that they did not receive much teaching that focused overtly on EHWB, regardless of whether a school had been identified as relatively high in EHWB provision or not. Several participants indicated that, where health related lessons did occur - delivered as part of the (non-statutory) PSHE curriculum - these tended to focus on other things:

Interviewer (I): do you have lessons on emotional health?

P1: ummm

P2: there's PSHE, it's usually drugs and that

P3: it's usually all the same thing about drugs or alcohol

P4: we've done cigarettes twice as well

P3: sometimes we do sex education, watching the same videos again and again

School 4 boys 12/13 years old

The staff interviews revealed a similar picture of a lack of EHWB lessons, although several individuals felt that they did cover emotional literacy. Five of the schools delivered PSHE as regular lessons, but in three cases all health education was limited to PSHE days or half days held a few times a year. In both scenarios, other topics tended to be prioritised:

When I think of the things we've been doing a lot of it is careers work, ummm, Industry Day, interviews, there's global awareness, fair-trade. There's not a massive amount on health and emotion. Oh, there's a friendship day in year 7. In year 9 we do an AIDS and relations game, in the past we've done 'no smoking' workshops

School 2 Head of Year

A second key theme from the student focus groups, was the inadequacy of EHWB lessons where they had been delivered, with participants feeling they did not cover useful topics and were not taught effectively:

P1: in citizenship sometimes they do stuff on bullying but it's not really that useful

P2: and it's just the same stuff over and over again

P1: yeah so maybe it would be good if there's like for a day or something someone comes in and we go in like small groups with your friends and they'd talk to you about how to stop bullies and stuff like that rather than just watching a tape or reading a book

School 6 boys 12/13 years old

Variations between but also within schools emerged as to how useful existing EHWB lessons were:

P1: some of them do actually teach you what you need to know but some of the others will be like

P2: just treat it like a free lesson

P3: others just read out what the lesson's about and then sit and do it. Most of them just do something else because they've no idea what to do

School 8 girls 13/14 years old

A possible reason for this emerged from the staff interviews. In six of the eight schools PSHE lessons were delivered by teachers or form tutors (a teacher assigned to each class who oversees its welfare, and monitors attendance, behaviour and support needs), who had mixed feelings about this role:

In this school it's delivered by tutors and for some people it's not what they would choose to be doing, it's not what they find easy to be doing. So in some instances people will prepare and take it seriously and teach it to the best of their ability and they might have an interest in it and they'll have some experience in various aspects and then for other people it's just a burden which they're not interested in so it's very last minute and the actual quality of children's experiences of those lessons will be quite variable

School 2 Head of year 8

A small number of students felt tutors were appropriate people to deliver EHWB lessons, but a key theme from the majority was that neither tutors nor teachers should teach this topic. This was either because of perceptions that teachers did not have the desire or expertise to do so, or because students felt uncomfortable discussing sensitive issues with them:

If you talk in front of a teacher they'll sort of always like be, probably every time they look at you you'll be thinking "oh no are they thinking about this, what I've talked about" and it'll be kind of awkward

School 8 girls 12/13 years old

Instead, students preferred the idea of having outside speakers with relevant training or experiences to lead EHWB sessions.

A majority of students felt that having more and improved EHWB lessons would be useful. They wanted such lessons to include activities, discussions, and IT, and for them to be conducted in a relaxed and informal way. Two groups suggested using an anonymous emotions box in which students placed an emotion or question relating to emotional health, which then formed the basis of a lesson. Some participants raised concerns that students may find speaking openly about their feelings difficult due to fears of being laughed at:

Some people would just laugh, like if someone had been bullied or the class doesn't like them and they get up and say something everybody would just laugh at them

School 3 girls 13/14 years old

For this reason many groups felt that small groups rather than whole classes would be the most appropriate format, and four of the 13 girls' groups suggested that EHWB sessions would best be conducted in single sex groups, due to concerns that boys would take the lessons less seriously, and would engage in teasing or disruptive behaviour.

The topics that students suggested should be covered were understanding feelings and how to express them appropriately, coping strategies for dealing with emotional distress, solving problems such as bullying and peer pressure, and accessing sources of help:

I think people don't know enough about that really like how to stop yourself feeling depressed or sad or something or how to make yourself feel happy

School 6 boys 12/13 years old

A few students suggested depression and self-harm as potentially useful topics to be covered, but others disagreed. A small minority were not in favour of any EHWB lessons, because they felt that it cannot be taught, they did not believe it to be relevant or they were concerned about the adverse effects of learning about such things:

P1: I don't want to think about people like cutting themselves and things

I: right, but do you think you don't need to know about it then?

P1: no, we already know

School 5 boys 13/14 years old

All staff interviewees also wanted to see more EHWB teaching, with lessons exploring coping strategies, understanding feelings, bullying, self-harm and bereavement mentioned in particular. However, three individuals raised the point that EHWB should not be seen as exclusively or even mainly a PSHE subject, believing it should pervade all aspects of teaching and school life, a suggestion which is returned to below:

I think it would be a complete and utter disaster if schools saw the word health in emotional health and thought oh that's PSHE isn't it, and so it gets stuffed over there. So I much prefer it to be a whole school thing... it's about the way you respond to and deal with people in your classroom, that's what the emotional curriculum should really be about

School 1 PSHE coordinator

Underpinning much of the student discussion about best formats for learning about EHWB within lesson time was a concern about stigma, both the stigma of experiencing emotional difficulty, but also the stigma attached to talking about emotions. This explained the concerns voiced by several students that they might be laughed at or treated differently if they contributed to EHWB lessons delivered by teachers or tutors, in whole class, mixed gender situations. Concerns over stigma led some students to comment on the value of other, non-health lessons such as English, Art and Sport, as potential places to learn about positive and negative emotional states more obliquely:

P1: you can build up anger in the day something might have happened and say you've got a rugby match after school you can let all your anger out then

P2: and you feel good about it

Voices: yeah

P3: it's like relaxation, even though you're getting munched

School 2 boys 12/13 years old

P1: the whole creative side like English, Art, Dance, Drama 'cause you can all express yourself through that sort of thing, but it doesn't necessarily have to be about you and people can't guess if it's about you or not so it's like expressing yourself without others knowing, that's what I find really good about it

School 8 girls 12/13 years old

Non-health related lessons therefore gave students the opportunity to discuss emotions impersonally, or to express feelings in anonymous ways, which avoided the possibility of being stigmatised.

#### Support for students in distress

The main themes that emerged in relation to schools supporting students experiencing distress were the importance of having someone to talk to, a lack of confidential, accessible and sympathetic help sources within schools, the importance of having easily accessible safe spaces within school to work through emotions, and the need to find non-stigmatising ways of providing support.

A majority of students discussed the importance of having someone to talk to when coping with emotional difficulty. In addition to teachers, a range of personnel within the schools existed who might be considered potential help sources, such as teaching assistants, who provide support to teachers within the classroom (in all 8 schools), learning mentors, who work with individual students to raise attainment (in 7 schools), a counsellor (in 6 schools), a chaplain (in 4 schools), a school nurse (in all schools) and a peer support service (in 7 schools). However, there was a great deal of variation between the schools in whether and when such people were available. Even in schools that appeared to provide a good amount of support, the students often did not know very much about the options available:

I: do you know much about the counsellor?

P1: I wouldn't know where to go, I think you go up to the Personal Guidance Centre I think

P2: who is the counsellor?

P1: I don't know much about it

School 4 boys 12/13 years old

Further, through the students' discussions of the various help sources, it became clear that they looked for particular attributes when evaluating potential help sources. Where students could approach help sources directly without anyone else knowing, and where they trusted them to maintain confidentiality, these tended to be the most popular. Other characteristics that emerged as important were having good availability and accessibility, being good at listening, being respectful of teenagers and their problems, and being able to provide useful information or advice. Just under one third of the girls' groups raised the gender of the help source as an issue, indicating that they would prefer help sources to be the same gender, although no boys' groups commented on this. Several students liked the idea of a help source being the same age because they were perceived to be more likely to understand and more trustworthy, but others argued that adults were more likely to have the information or knowledge necessary to help.

Conversely, several barriers to seeking help from these various sources emerged. Some of these related to characteristics that were attributed to the help source, in particular being difficult to access, being unable or unwilling to help or being unlikely to be trustworthy. Other barriers related to perceived consequences of seeking help, such as not being taken seriously, being treated differently in the future, or exacerbating the problem. Many of the students' comments about the negative consequences of help-seeking related to fear of being stigmatised, which was partly why students emphasised the need for help sources to be confidential, and able to be accessed without anyone else knowing. These concerns also led to some students feeling they would prefer a help source to be a relative stranger, echoing some of the concerns regarding EHWB lessons:

And sometimes talking to the head of year isn't really good because you see your head of year every day so sometimes you just want to talk to somebody you don't see every day because [*pause*] it's like you don't want to be treated differently, you want to talk about it but you want to be treated the same anyway. You want to be normal basically, everybody wants to be normal

School 6 girls 13/14 years old

Of all possible school-based help sources, the chaplains where they existed appeared to meet most of the desired criteria most of the time, and therefore received the most favourable reaction:

P1: you can just go up to the desk and ask for them [the chaplains] and also it's better than going to teachers 'cause if it's a teacher it might sometimes affect your lessons and you hardly ever see them anywhere and you know it's not going to affect the rest of your school life

P2: they don't just sit there taking notes and have a glazed look over their eyes 'cause they're not actually listening

P1: yeah and they don't go "and how do you feel about that" they just talk to you like you're a normal person

School 8 girls 12/13 years old

Learning mentors, counsellors and teachers had a mixed reaction, with some students regarding them as helpful, whereas others expressed concerns that they would not keep problems confidential, that they were not be understanding, or that they were not available to everyone enough of the time. School nurses were not regarded positively by any of the seven groups that suggested them as a possible help source, due to perceptions that they were unavailable, or were only interested in physical illness. Similarly, the peer support schemes, when mentioned, engendered a negative response from most students in the schools where they existed, due to concerns that the service would not be confidential, that the peer supporters were only the clever or popular students who would not understand, that they might laugh at the person asking for help, and that they were unlikely to be able to help:

P1: you talk to them and then they probably go off and laugh about you and have a discussion

P2: they don't know anything do they? They're just children

P3: they get no training whatsoever, they just give it to the clever people

School 5 boys 13/14 years old

Students felt that a priority for schools should be having more help sources with the right attributes available. Although staff were generally more positive about the help available, some agreed that more was needed:

Before we were saying our job is learning support you know and we're supposed to be in the lesson but 90% of my time is listening to a child it could be something going on at home, at school, anything...there is just not enough of us, you know, I mean I'm on dinner duties as well a couple of times and they could literally be queuing you know to talk to you, um because you're there and you're a face and they just need someone to talk to

School 6 Teaching Assistant

A few students also suggested that schools could do more to assist them in accessing external help sources, for example by providing leaflets and posters. All the schools displayed various posters relating to mental health, but one student pointed out this was not always helpful, due to concerns of being seen by others standing in front of a poster trying to memorise a phone number. One school avoided this problem by putting helpline numbers in the back of homework diaries, which all students had to keep with them.

As well as having access to appropriate help sources, some students felt strongly that there was a need for safe spaces, where students could relax away from difficult situations, or be left alone to process emotions such as anger, without other students or staff attempting to intervene:

P1: I don't know how to say it but have like a room where you can let your anger out

P2: somewhere like you can let off steam

P1: yeah

School 7 boys 13/14 years old

In some of the schools, staff did discuss separate areas or buildings that were intended to function as a safe haven for those feeling vulnerable, and a place to access help sources. However, the student participants did not always know much about these places where they existed, and access was generally monitored and controlled by the staff involved, therefore they did not provide the more informal and anonymous 'chill out' spaces that the students said they wanted.

The other main support mechanism identified by staff and students, provided in all eight schools, were support groups for 'vulnerable' individuals. On the whole, students felt that these were a good idea, although there were some concerns expressed that the selective nature of such groups could lead to some students missing out, or those attending being stigmatised:

P1: people will use it against you and like cuss us or something and say "ah you have to go to your stupid emotional health thing"

P2: you'll feel like you're special needs and they'll say like "well at least I'm not dumb and at least I'm not special needs"

School 6 boys 12/13 years old

One staff member described a support group in her school that avoided these problems by being open to everyone, and that while originally set up as an exam support group, had successfully evolved into one that dealt with any emotional health issues that were raised. Two student groups suggested the idea of student-led groups, following a self-help model, but this was not currently provided by any of the schools.

#### The School Environment

Much of the discussion in the staff interviews and student focus groups highlighted the importance of the school environment, both physical and psychosocial, in impacting on student emotional health. Key themes that emerged were the sources of distress that existed in the school context - specifically bullying from peers, difficult relationships with teachers and academic work - and the potential of schools to bolster student emotional health through whole-school changes such as providing a supportive culture and improving the physical environment.

All of the focus groups discussed ways in which schools had a negative impact on emotional health, with three key sources of distress that were raised directly relating to school-life. Bullying was identified as a major cause of emotional distress by 23 focus groups, with the majority of participants feeling that schools did not do enough to deal with it effectively:

P1: if you do tell the teachers they usually say they'll do something about it and then nothing happens

P2: or they go up to them and have a word with them and then it just carries on it has no effect at all

P1: they don't really follow anything through

School 8 girls 12/13 years old

Several staff members also saw bullying as a significant problem, and outlined various ways in which their schools had attempted to address this, such as poster competitions, a focus on bullying in lessons and assemblies, and an ongoing anonymous e-mail and texting service to report incidents. However, only one school appeared to have been successful in the students' eyes, with participants at this school less likely to see bullying as a problem than those in the other schools.

A second main source of emotional distress, identified by 25 of the 27 student focus groups, was problematic relationships with certain teachers, with students describing unfair, unhelpful or even humiliating treatment:

P1: she [teacher] says like "I'll pick you up by your sidies [sideburns] and throw you out of the window"

I: how did that make you feel?

P2: like an idiot

P1: yeah

P3: ashamed

P1: yeah

School 5 boys 12/13 years old

Even where teachers were not the source of emotional distress, some students noted that their lack of sympathy to students' difficulties could make matters worse:

P1: sometimes if things are happening outside of school that teachers don't understand and then they have a go at you anyway it just makes it worse

P2: Yeah that's true, things will happen at home with me and then I come to school and the teachers will shout at you and you feel so frustrated and you tell them what's happened at home and they still wouldn't, oh that's no excuse

School 6 girls 13/14 years old

Staff interviewees did not express staff-student relationships quite so negatively, but some agreed that colleagues could contribute to students' emotional distress:

You know a lot of problems are caused by just a throw away remark, and it's not meant to be malicious or anything like that but that child takes it away with them and it makes them feel as though they're not wanted and that can be done by saying "oh it's good of you to drop in" or something like that you know? The teacher doesn't mean it nastily, but if that's someone that's found it difficult to get into the classroom"

School 3 Learning Support Manager

Finally, being overburdened with academic work and exams were mentioned by more than half the student focus groups as a source of emotional difficulty. Some staff agreed:

I think some of our youngsters are coming in to school dealing with all sorts of issues which I as a child I never had to deal with and they're really burdened with things, some are young carers for example, and then we're expecting them to work quite a long day and under a great deal of pressure and I do think we need in our curriculum time to give them some creative time or relaxation time

School 3 Assistant Principal

Conversely, the importance of school as a place where emotional health can be enhanced, or at least emotional distress relieved, was a theme that emerged from several staff and students' discussions:

I feel one of our selling points is that we offer very much a caring environment, we might not have the 49 sports halls but we know every student that walks through the door

School 8 Head of Year 7

But some people bring it to school 'cause they can't bring it anywhere else, they have so much anger it's like they can't take it out anywhere else

School 3 girls 12/13 years old

Students made several suggestions about changes that could be made, in order to achieve a physical and psychosocial environment that supported emotional health in this way. Key ideas were increasing the amount of rewards and praise for good behaviour, making lessons more enjoyable through activities and use of IT, having more extra-curricular activities, improving the physical environment, and being less strict about what students regarded as trivial matters such as having the correct uniform. Suggestions from staff echoed some of these views, in particular having more encouragement and attention for students who behave well, providing a wider range of activities during breaks, and improving the physical environment:

We're looking at students being safe and enjoying the school, we need picnic tables so they can sit down and eat their sandwiches in the summer when it's nice, in the shade. We need some basketball nets and netball nets out in the courts, they need safe areas, an activities club for the lower school where students who aren't sporty who want to sit down and play chess games who just want to do some crafts can go, it's at lunchtime so they're safe and they know it's somewhere they can go

School 5 Head of Key Stage 3

## Discussion

### Main Findings

The school is frequently identified as an important setting for addressing emotional health [15,26,34]. The participants of this study echoed this view, through their identification of a number of ways in which this could be achieved. However, several gaps were revealed between what students and staff perceive as important and what is currently provided.

All participants agreed that very little lesson time was spent on EHWB, with comments indicating that other topics, notably sex and drugs, were more commonly taught. It could be argued that emotional health/distress underpins much of the behaviour and decision-making relating to drug use and sexual activity, and indeed the survey indicated that more work was being done on emotional health in lesson time than the respondents appeared aware of, which may be due to these links with other curriculum content. Nevertheless, it is noteworthy that many participants wanted more lessons that dealt overtly with emotional health, covering topics such as understanding feelings, coping with emotional distress and accessing help sources. A minority of students did have reservations about this idea, and several voiced concerns about the stigma attached to experiencing or even discussing difficult emotions, suggesting instead that emotional health lessons should be delivered in small groups, by outside experts rather than teachers. An equally important finding is therefore the way that other, non-health related curriculum subjects could provide students with a safer context in which to explore and learn about EHWB.

Students and staff also felt that schools should provide better support for those in distress, for example through someone to talk to, support groups and safe spaces for relieving emotions and receiving help. Although the survey data indicate that a majority of schools currently provide some form of support for students in need, the qualitative data revealed great variety in the number and quality of those services. Students did not always have a favourable opinion of the support that existed, particularly if it was not perceived to be confidential, available to all or sympathetic to the needs of teenagers. A key concern was stigma, and fear of being seen or treated differently, which could create a significant barrier to a help source being used.

Finally, several participants emphasised the importance of the school environment in supporting or damaging students' emotional health. A clear message was therefore that any EHWB work had to include consideration of the physical and psychosocial environment. Suggestions for changes in this regard included reducing bullying, improving teacher - student relationships, increasing rewards and recognition of good behaviour, and developing the range and number of extra-curricular activities available.

### Strengths and Limitations

This is the largest study to have qualitatively explored staff and student views of EHWB provision in secondary schools. The background survey data provided a useful indication of the sorts of provision that is currently delivered in schools, but the in-depth qualitative findings made a much more detailed and contextualised picture available regarding the variability, acceptability and usefulness of those activities from the perspective of those at whom they are targeted. In fact few differences between schools designated as providing high levels of EHWB activity and those labelled as low level emerged in the qualitative data, which may be explained by the different levels of detail that each data collection method allowed. For example, the survey asked whether a school provided an onsite counsellor or not, to which 80% of respondents answered yes. However, the qualitative data revealed several problems with this provision from the viewpoint of participants, such as the counsellor not being in the school for many hours, students not being able to self-refer, and students not knowing about the service or how to access it. Such findings highlight the value of gathering qualitative data when assessing what schools can do to support emotional health, in order to explore the quality and appropriateness of the provision from the perspective of the users.

The fact that the focus of the study was on students' and staffs' views means the findings may not be a comprehensive reflection of all the ways in which schools do support emotional health. For example emotional health may be covered more obliquely in the PSHE curriculum as it relates to topics such as drugs and sex and relationships. However, by focusing on the views of those involved, this study was able to identify discrepancies between what students *were aware *they were receiving in terms of emotional health support and education, and how far they felt this measured up to what they would find useful. A further strength was the ability to compare students' perceptions with those of staff involved in delivering EHWB provision - although there were some disparities regarding what staff said was available and what students were aware of, there were striking similarities in terms of the improvements that both groups suggested could be made.

One limitation of the study is that because teachers selected half the participants for the focus groups, it is possible that an element of "gatekeeping" went on. For example it is possible that students were more likely to have been selected if they were expected to portray the school favourably, or if they were known to have experienced emotional distress or made use of certain services. A second potential limitation is that by conducting student focus groups rather than individual interviews, an element of group conformity may have been introduced, for example students who had more positive views of provision in their school may have felt unable to express this. The groups were single sex and made up of friendship pairs in an attempt to encourage students to speak openly, and in fact the many examples of students disagreeing and contradicting each other, suggested this group effect was limited. Further, using focus groups allowed students to comment on and add to each other's views, creating richer and more detailed accounts than might have been gathered in a one to one situation.

### Implications for Policy and Future Research

The fact that very little lesson time is spent on EHWB means that key areas relating to emotional health such as understanding feelings, depression and self-harm are neglected by a majority of schools. Evidence that young people downplay symptoms of emotional distress and delay accessing formal help sources [47,57] indicates that topics such as understanding and coping with difficult feelings, and knowing when and how to seek help could usefully be addressed within lesson time. Although there is some promising evidence that both preventative and educational classroom interventions might be effective at improving knowledge and skills related to emotional health and distress [17,23], studies with more robust designs are needed to establish the most useful ways to approach this long term. Such studies must also explore the potential for harm to which explicit teaching about EHWB might lead. A minority of participants did not want such lessons to be introduced, not least because of the stigma associated with disclosing emotions and emotional distress. Further, emotional literacy programmes such as SEAL in England have been criticised for imposing a particular way of experiencing and expressing emotions on all young people, and disempowering individuals by positioning them as emotionally vulnerable in the first place [58]. Given such concerns, the point raised by several students in this study that creating greater opportunities for exploring emotions more anonymously in other lessons such as English and Drama, should also be acknowledged and supported in policy documents.

The students and staff made several insightful comments regarding the best format for EHWB lessons, which should be explored in future research, for example whether small and informal groups would result in more valuable EHWB lessons. The question of whether such lessons should be single-sex should also be examined, both in terms of whether the views expressed here by some female participants are shared by other students, particularly males and other age groups, and also in terms of any evidence that single-sex or mixed-sex groupings are more effective. The point made by some girls here that boys would be disruptive echoes a similar debate within the sex education literature. Here, it has been suggested that single-sex lessons are desired by many students due to embarrassment of both girls and boys in talking openly in mixed classes, reports of disruptive and demeaning behaviour of boys towards girls, and differing needs in terms of content [52,59], although others have challenged these points [60,61].

Another key question raised by the students' preferences reported here is whether using outside experts is more effective than using teachers, and whether this is sustainable long term. There is some evidence from other areas of health education that involving outside speakers is something many students want [61], and is something that can be effective as a supplement to a school's ongoing educational programme [62]. However, concerns have been raised that there can be practical difficulties in organising this, and that 'cultural clashes' can arise in terms of key messages [63]. Two published studies have examined the involvement of external contributors in mental health interventions in English secondary schools and, although showing promising results, neither had control groups with which to compare the results [20,64]. Collaboration between schools and external agencies would help identify outside experts within the local context who might be willing to contribute to lessons [65]. Indeed where schools have counsellors or other emotional health support staff based in schools, they too could play a role. However, the practical and financial implications of involving external experts makes it likely that teachers would still play a role in delivery, meaning better EHWB training, and specialist teaching teams in every school, are essential.

Several extra-curricular changes also need to be considered. The finding that many students consider having someone to talk to within school to be an important source of support resonates with previous research indicating that school-based counselling services are effective and regarded positively by students and staff [66]. However, the schools visited here varied a great deal in the amount and type of help available. A key issue that emerged was the need to provide help sources that fulfilled certain criteria, in particular that were confidential, easily available, good at listening, respectful of teenagers and their problems, and able to provide useful information or advice. These features resonate with previous findings regarding students' views of school-based help sources [67,68], as well as with the views of adolescents regarding previous experiences with 'helping professionals' more generally [69]. Such findings underline the importance of ascertaining students' views of services, to ensure they have a correct understanding of what they provide, but also to identify areas where provision can be improved to better meet their needs. The peer support schemes were a striking example of this; such interventions have become very popular among practitioners over the past decade [70] - indeed the survey revealed that over 80% of schools provided such a service - but this favourable view was not shared by the students in this study. Conversely, where chaplains existed, they did appear to match the criteria that were important to students. Generally it is only schools with a religious affiliation that would have regular access to a chaplain, however more exploration of this role - what it is about the way chaplains operate or behave that enables them to fulfil the desired criteria - may help identify a blueprint useful for all schools in providing the most effective forms of support possible.

A key concern raised by students was that some of the support offered - for example support groups for 'vulnerable' students, and non-confidential help sources - could lead to stigma and students in need missing out on help. The relative value of targeted versus universal approaches in the field of adolescent emotional health is yet to be clearly established [71], but it is noteworthy that the student views reported here emphasise an approach that allows confidential access to interventions for all individuals. One such intervention that might be easily introduced and that appeared popular with participants was a 'chill out' area, in which students could be left alone to work through their emotions.

In addition to the introduction of specific educational and supportive interventions, students and staff felt strongly that changes to the whole school environment needed to be considered, due to the potential of schools to contribute to emotional distress or to bolster emotional health. This view resonates with the emphasis on whole-school approaches to tackling emotional health in some studies, and in most policy documents in England [25-27,34]. The three aspects of the school context that students identified as causing the most emotional distress - bullying, difficult relationships with teachers and academic stress - have all been found to be detrimentally associated with mental health [30,72,73], and would be useful areas for schools to target, for example through developing relationship policies, or providing workshops focused on coping with exams. Conversely, staff and students identified several areas for change that might enhance emotional health, including greater recognition for good work and behaviour, a pleasanter physical environment, and a greater range of extra-curricular activities. The survey revealed that only just over a quarter of the responding schools provided any EHWB training in general for teachers and this would need to be increased, if teachers are to understand the school-based causes of emotional distress, and ways in which emotional health can be better supported through day to day school life and interactions. These findings indicate the need to avoid introducing additional EHWB lessons or support services in isolation, but to take a more holistic approach, in which consideration is given to current policies and practices throughout the school, and ways in which these can be made more supportive of student emotional health [74].

## Conclusion

Although all the schools within this study were delivering some form of EHWB work, students and staff agreed that much more needs to be done in terms of teaching about emotional health within lessons, provision of appropriate support, and improving key aspects of the physical and psychosocial environment. Such changes require greater clarity at policy level in terms of what schools, in collaboration with external agencies, should provide in this area. Further, more rigorous evaluation is needed to determine what initiatives are effective, so examples of effective practice can be implemented more widely. Improving emotional health provision in schools is vital for supporting the emotional health of all teenagers, and for intervening early to reduce emotional disorder and associated detrimental outcomes among this age group.

## Competing interests

The authors declare that they have no competing interests.

## Authors' contributions

JK conceived of the study, participated in its design, conducted the data collection and analysis, and wrote the paper. JD contributed to the study design, data analysis and interpretation, and revision of the manuscript. LB contributed to the data collection, analysis and interpretation, and revision of the manuscript. RC contributed to the data analysis and interpretation, and revision of the manuscript. DG contributed to the study conception and design, data analysis and interpretation, and revision of the manuscript. All authors have read and approved the final manuscript.

## Pre-publication history

The pre-publication history for this paper can be accessed here:


